# A systematic review of qualitative findings on factors enabling and deterring uptake of HIV testing in Sub-Saharan Africa

**DOI:** 10.1186/1471-2458-13-220

**Published:** 2013-03-11

**Authors:** Maurice Musheke, Harriet Ntalasha, Sara Gari, Oran Mckenzie, Virginia Bond, Adriane Martin-Hilber, Sonja Merten

**Affiliations:** 1 Zambia AIDS-related TB Research Project, University of Zambia, P.O Box 50697, Lusaka, Zambia; 2 Swiss Tropical and Public Health Institute, Socinstrasse 57, Basel, CH-4002, Switzerland; 3 University of Basel, Faculty of Science, Petersplatz 1, Basel, CH-4003, Switzerland; 4 Department of Social Development Studies, University of Zambia, Lusaka, Zambia; 5 Department of Global Health and Development, Faculty of Public Health and Policy, London School of Hygiene and Tropical Medicine, Keppel Street, London, WC1E 7HT, U004B

**Keywords:** HIV, HIV testing, Antiretroviral therapy, Meta-ethnography, Sub-Saharan Africa

## Abstract

**Background:**

Despite Sub-Saharan Africa (SSA) being the epicenter of the HIV epidemic, uptake of HIV testing is not optimal. While qualitative studies have been undertaken to investigate factors influencing uptake of HIV testing, systematic reviews to provide a more comprehensive understanding are lacking.

**Methods:**

Using Noblit and Hare’s meta-ethnography method, we synthesised published qualitative research to understand factors enabling and deterring uptake of HIV testing in SSA. We identified 5,686 citations out of which 56 were selected for full text review and synthesised 42 papers from 13 countries using Malpass’ notion of first-, second-, and third-order constructs.

**Results:**

The predominant factors enabling uptake of HIV testing are deterioration of physical health and/or death of sexual partner or child. The roll-out of various HIV testing initiatives such as ‘opt-out’ provider-initiated HIV testing and mobile HIV testing has improved uptake of HIV testing by being conveniently available and attenuating fear of HIV-related stigma and financial costs. Other enabling factors are availability of treatment and social network influence and support. Major barriers to uptake of HIV testing comprise perceived low risk of HIV infection, perceived health workers’ inability to maintain confidentiality and fear of HIV-related stigma. While the increasingly wider availability of life-saving treatment in SSA is an incentive to test, the perceived psychological burden of living with HIV inhibits uptake of HIV testing. Other barriers are direct and indirect financial costs of accessing HIV testing, and gender inequality which undermines women’s decision making autonomy about HIV testing. Despite differences across SSA, the findings suggest comparable factors influencing HIV testing.

**Conclusions:**

Improving uptake of HIV testing requires addressing perception of low risk of HIV infection and perceived inability to live with HIV. There is also a need to continue addressing HIV-related stigma, which is intricately linked to individual economic support. Building confidence in the health system through improving delivery of health care and scaling up HIV testing strategies that attenuate social and economic costs of seeking HIV testing could also contribute towards increasing uptake of HIV testing in SSA.

## Background

HIV continues to be a public health burden in sub-Saharan Africa (SSA). Out of an estimated 34 million people living with HIV worldwide at the end of 2010, 68% resided in SSA [1] and an estimated 1.9 million people became newly infected in 2010 [2]. Efforts to achieve zero new infections and zero AIDS-related deaths [3] require increased uptake of HIV testing as a gateway to HIV prevention, treatment and care. To address this health problem, coupled with increasingly wider availability of antiretroviral therapy (ART), many countries in SSA have in recent years dramatically scaled up HIV testing services. For instance, health facilities providing HIV testing services in 37 countries of SSA increased by 50% from 11,000 in 2007 to 16,500 in 2008 [4].

Despite the increasingly wider provision of HIV testing services, ten population-based surveys estimate that the median percentage of people living with HIV who know their status is below 40% [5]. Quantitative studies have identified stigma and discrimination [6]; perceived low risk of HIV infection [7]; perceived lack of confidentiality [8]; and distance to testing sites [9] as barriers to uptake of HIV testing. Enabling factors include perceived anonymity of testing [10]; convenience of home-based HIV testing [11]; and availability of ART [12]. Qualitative studies have also been conducted in SSA that additionally highlighted social dynamics influencing uptake of HIV testing. Despite the volume of this evidence and the contribution it can make towards a better understanding of factors influencing uptake of HIV testing in SSA, systematic reviews are lacking.

## Methods

We used the meta-ethnographic approach first put forward by Noblit and Hare [13] to synthesise published qualitative research findings. Meta-ethnography has increasingly been used to re-interpret and synthesise qualitative research findings across multiple studies in order to gain in-depth understanding of a phenomena [14-17]. This involves the ‘juxtaposition of studies and the connections between them’ [18] in order to achieve greater conceptual development and insight than would be obtained from individual studies [15]. Emphasis is on developing new interpretations and concepts rather than accumulation of information [19].

### Search strategy and identification of papers

**Figure 1:**
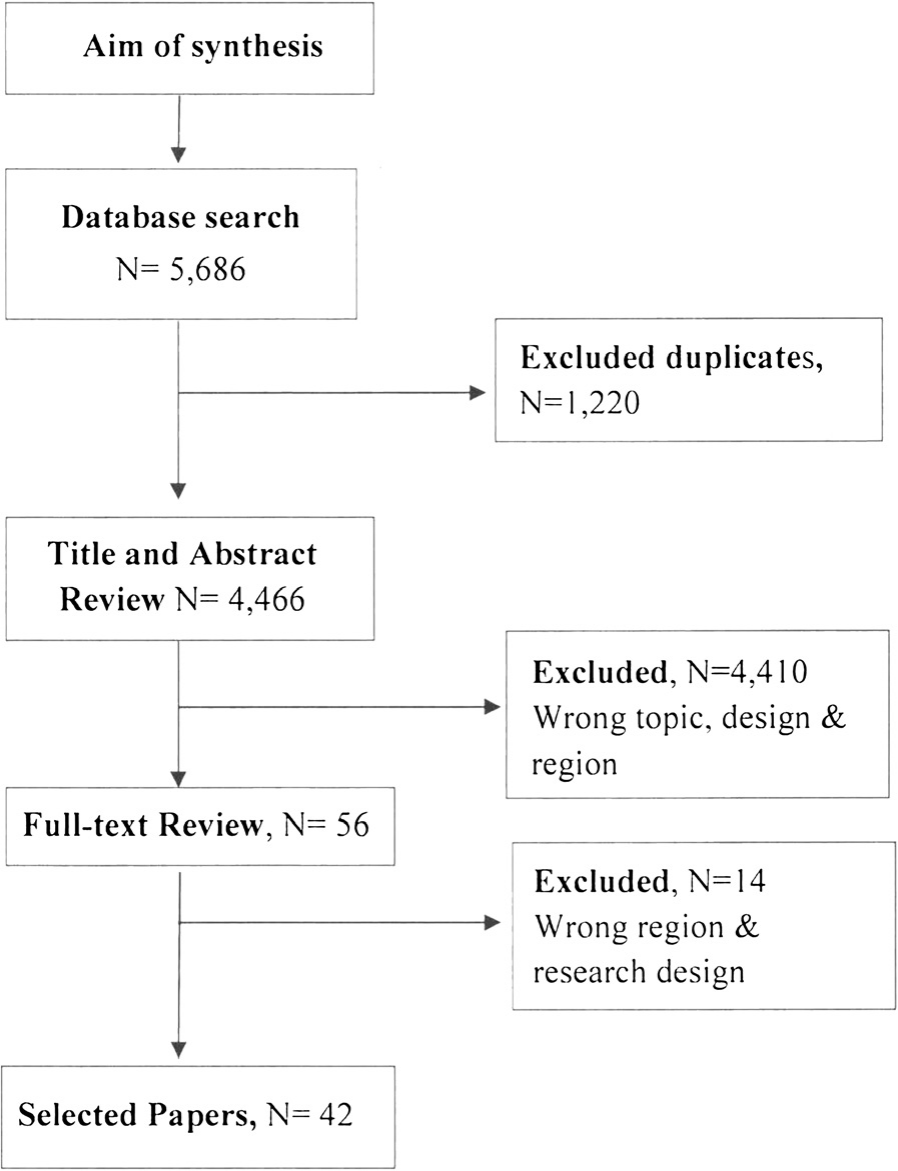
Search strategy and paper selection flowchart.

CINAHL, CSA, EMBASE, JSTOR, Medline and Web of Science were searched for published qualitative research findings up to end of February 2012. The first search was done on 30^th^ June 2010 and repeated on 26^th^ February 2011. Repeated searches using the same search strategy were undertaken until end of February 2012 to ensure that no new publications were omitted. The searches yielded 5,686 citations of which 4,466 were subject to title and abstract review. 1,220 were duplicate papers. An over-inclusive search strategy was used to ensure that no papers were missed. The key search words used were: “HIV OR “Acquired Immuno-deficiency Syndrome” OR AIDS OR “HIV infection” or “HIV/AIDS”; VCT OR “voluntary counselling and testing” OR “HIV test”; Africa OR “sub-Saharan Africa” OR SSA. We also reviewed references of the selected papers to ensure that no papers were missed. Two researchers (MM and HN) reviewed titles and abstract in duplicate to exclude ineligible articles. Papers that met the inclusion criteria were subject to full-text review (Figure 1).

### Quality assessment and inclusion criteria

Quality appraisal of qualitative research still remains contested [20-23] because ‘there is no unified body of theory, methodology or method that can collectively be described as qualitative research’ [24]. Previous meta-ethnography studies have not used any formal appraisal checklist [17,20] or did not exclude any paper on the basis of pre-specified quality assessment criteria [22,25,26]. Barbour [27] has pointed out that while checklists are useful in improving qualitative research, such ‘technical procedures’ can affect the contributions of systematic qualitative research. With this contestation in mind, but to ensure inclusion of relevant ‘quality’ papers, our inclusion criteria comprised: peer-reviewed publications only; published in English; conducted in SSA; focused on access to HIV testing; and reported qualitative findings - including mixed-methods papers. Forty-two (42) publications from thirteen (13) SSA met the inclusion criteria (Table 1).

**Table 1:** Characteristics of selected papers

**1**^**st**^**Author/Year [Citations]**	**Country**	**Settings Urban/Rural**	**Sample (sub-group)**	**Study type**	**Aim**
Castle, 2003 [28]	Mali	Urban	Men & women 20–34 yrs; Young people 17–24 yrs	Qualitative	To assess attitude towards HIV with a view to setting up VCT services.
Pool, 2001 [29]	Uganda	Rural	Women antenatal care attendees	Qualitative	To explore attitudes to VCT among women attending antenatal care.
Daftary, 2007 [30]	South Africa	Urban	In-patient with TB	Qualitative	To explore decision making processes for HIV testing and disclosure by TB patients.
Maman, 2001 [31]	Tanzania	Urban	Men, women, Couples	Qualitative	To explore individual, relational and environmental factors influencing HIV testing decision & disclosure of status to partners.
Mabunda, 2006 [32]	South Africa	Rural	Men and women ≥ 18yrs	Qualitative	To identify themes related to VCT services in rural South Africa.
Angotti, 2009 [33]	Malawi	Rural	Married women & men 15–49 yrs; Married & unmarried adolescents 15–24 yrs	Qualitative	To examine the acceptability of HIV testing in 3 rural districts.
MacPhail, 2008 [34]	South Africa	Urban	Adolescents 12–24 yrs; parent	Qualitative	To establish the perceptions of and needs for VCT among young people.
Mlay, 2008 [35]	Tanzania	Urban	Women 18–49 yrs; Men 20–75 yrs	Qualitative	To gain insight into the views of counsellors men and women on VCT for couples.
Izugbara, 2009 [36]	Malawi & Uganda	Rural/Urban	Male youths 14–19 yrs	Qualitative	To offer youth-centred perspectives & masculinity as they relate to HIV services, including VCT.
Grant, 2008 [37]	Zambia	Urban	People living with HIV	Qualitative	To examine what factors affect a person’s decision to seek testing and then start and stop treatment.
Denison, 2008 [38]	Zambia	Urban	Adolescents 16–19 yrs	Qualitative	Explore how adolescents involve their families, friends, sex partner about VCT & disclosure of status.
Oshi, 2007 [39]	Nigeria	Urban	University Students	Qualitative	To investigate if self-perception of risk of HIV infection causes Nigeria youths to reduce risky sexual behaviour & seek VCT.
Meiberg, 2008 [40]	South Africa	Urban	University Students	Qualitative	To identify psychosocial correlates of HIV voluntary counselling & testing.
Groves, 2010 [41]	South Africa	Urban	Women at antenatal clinic	Qualitative	To explore women’s experiences with HIV testing & the consent process in a public antenatal clinic.
Råssjö, 2009 [42]	Uganda	Urban	Young men & women	Qualitative	Attitude to VCT among young men & women in a slum area of Kampala, Uganda.
Chirawu, 2010 [43]	Zimbabwe	Rural	Men and women ≥ 18yrs	Mixed methods	To examine the acceptability & feasibility of providing client-initiated VCT in health facilities & research-initiated VCT in a non-clinic setting.
De Paoli, 2004 [44]	Tanzania	Rural	Women at antenatal clinic	Mixed methods	To identify factors associated with pregnant women’s willingness to accept VCT.
Ayenew, 2010 [45]	Ethiopia	Not stated	Patient with TB; Nurse counsellors	Mixed methods	To assess predictors of HIV testing among TB patients.
Namakhoma, 2010 [46]	Malawi	Urban & Rural	Health Workers	Mixed methods	To explore the enablers and access barriers to HIV-VCT & ART by health workers in Malawi.
Urassa, 2005 [47]	Tanzania	Not Stated	Women 15–45 yrs old	Mixed methods	To identify risk factors for preferring to avoid HIV testing among women attending antenatal care.
Obermeyer, 2009 [48]	Burkina Faso	Urban & rural	Men & women	Mixed methods	To investigate the utilization of services around HIV testing.
Bhagwanjee, 2008 [49]	South Africa	Not stated	Mine employees	Qualitative	To understand users’ perceptions of VCT & HIV treatment services offered by a mining company.
Luginaah, 2005 [50]	Ghana	Not stated	Pastors, Marriage counsellors, men & women	Qualitative	To examine efforts by some men & women churches in Ghana to reduce the spread of HIV through HIV-VCT.
Taegtmeyer, 2006 [51]	Kenya	Urban & rural	Men & women	Mixed methods	To better understand the reasons behind gender differences in Kenyan VCT sites.
Larson, 2010 [52]	Uganda	Urban	Men	Qualitative	To explore men’s views on and experiences of couple HIV testing during antenatal care.
Varga, 2008 [53]	South Africa	Urban & rural	adolescent mothers 15–19 yrs old	Qualitative	To examine barriers to HIV testing uptake & participation in PMTCT services.
Sherr, 2003 [54]	South Africa	Urban & rural	Health staff & women	Qualitative	To establish the attitude of clinic staff & pregnant women to routine HIV testing & counselling.
Simpson, 2010 [55]	Zambia	Urban & rural	Cohort of school boys	Qualitative	To describe masculinity, religious ideas & response to VCT among a cohort of catholic boys.
Nuwaha, 2002 [56]	Tanzania	Urban & rural	Men & women	Mixed methods	To understand factors influencing choice of VCT.
Theuring, 2009 [57]	Tanzania	Rural	Men of reproductive age	Mixed methods	To assess male attitude regarding partner involvement in ANC and PMTCT services.
Mbonye, 2010 [58]	Uganda	Rural	Women, Men & adolescents Local leaders & health workers	Mixed methods	To understand care-seeking practices and barriers to PMCT services.
Levy, 2009 [59]	Malawi	Urban	HIV-positive women; PMTCT programme managers; policy makers; health workers	Qualitative	To examine women’s decisions about HIV testing & experiences of PMTCT & HIV-related care.
Bwambale, 2008 [60]	Uganda	Rural	Men & women aged ≥18 years; CHWs; NGO health workers	Mixed methods	To determine the prevalence and factors associated with VCT use amongst men.
Frank, 2009 [61]	Zambia	Rural	Women and men with HIV; village leaders; health workers	Qualitative	To determine if community structures and livelihood strategies were changing to mitigate the impact of the HIV epidemic.
Larson, 2012 [62]	Uganda	Rural	Pregnant women at ANC	Qualitative	To explore pregnant women experiences of opt-out HIV testing.
Dye, 2011 [63]	Kenya	Rural	Men & women in the community	Qualitative	To ascertain motivational & experiential dimensions of participation in rapid integrated prevention campaigns.
Roura, 2009 [64]	Tanzania	Rural	Community leaders, ART users, Health workers	Qualitative	To investigate the effects of ART scale up on stigma & HIV testing in rural Tanzania.
Day, 2003 [65]	South Africa	Urban & rural	Mine workers	Mixed methods	To identify the attitude influencing uptake of VCT among Gold mine workers in South Africa.
Phakathi, 2011 [66]	South Africa	Rural	Community members	Qualitative	To examine the influence of ART on willingness to test for HIV in a rural community.
Skovdal, 2011 [67]	Zimbabwe	Rural	ART users, Health workers, care givers of children on ART	Qualitative	To examine how local construction of masculinity impact on men’s use of HIV services.
Njozing, 2010 [68]	Cameroon	Not stated	TB Patients	Qualitative	To explore the barriers and barriers to HIV testing among TB patients.
Jürgensen 2012 [69]	Zambia	Urban & rural	Community members & VCT counsellors	Qualitative	To explore local meaning attached client initiated HIV testing in rural & urban setting of Zambia.

### Analysis and synthesis process

To establish how the concepts from different papers were related to one another, we created a grid and entered the concepts from each paper (see Table 2). We used Malpass’s notion of first-, second-and third-order constructs to generate the concepts [26]. First-order constructs represent the views of research participants while second order-constructs are authors’ interpretation of research participants’ views [14,23]. Second-order constructs were identified, cross-compared and used to develop third-order constructs - our interpretations of the researchers’ interpretation of research participants’ views [23,26].

**Table 2:** Translation table of factors influencing uptake of HIV testing

**3**^**rd**^**Order constructs**	**2**^**nd**^**Order constructs**	**Summary definition (translation) of 1**^**st**^**& 2**^**nd**^**order constructs**	**Source Papers**
Lay construction of risk of infection & health.	Low self-perception of risk of infection.	Perception of being at less risk of infection and carrying on with life as normal sometimes based on HIV status of sexual partner.	[36,38,43-45,49,50,56,60,67]
	Chastity/sexual inactivity.	The lack of a sexual partner or abstinence from sex creates perception of being at less risk of infection.	[38,50]
	Ill-health &/or death of child/sexual partner.	Experience of sexually transmitted infection, physical deterioration of one’s health or poor health/death of sexual partner/child creates a sense of susceptibility to HIV infection.	[30-33,37,39,40,42,43,48,49],[55,56,59,60,65,67-69]
	Social contact with person with HIV.	Personal contact/knowing someone with HIV or who had died of AIDS raise concern about its existence & susceptibility thus creating a sense of vulnerability. In settings with low prevalence creating a sense of vulnerability. In settings with low prevalence HIV raises doubts about its existence, and creates social distance from HIV.	[28,40,49]
	Risky sexual lifestyle.	Experience of multiple sexual partners, including past sexual life or perceived partner infidelity either creates a sense of susceptibility or creates assumption of already being infected.	[32,34,36,39,40,42,44,48],[59,60,69]
Mental burden of living with HIV.	HIV+ status as imminent death & psychological burden.	In the absence of a cure, despite the availability of ART, HIV positive status perceived as hastening death. Thus, imminent death is avoided by shunning HIV testing.	[29,33,34,37,38,40,42,44],[47,48,53,55,57,60,65,68],[69]
		Perceived incapacity to psychologically cope with a positive HIV result & associated lack of will to live with HIV.	[32,34,36,40,43,45,46,48],[53,54,56,61,69]
Social support & exclusion.	Family & peer network influence & support.	Social influence and green light from family and friends influence decision making (not) to test.	[36-38,40,42,47,56-58,67]
		The fears of losing social/economic (support) networks, including sexual partners discourage HIV testing.	[38-40,42,64,67]
	(Mis-) trust in marital relationships.	In marital relationships with perceived mutual trust & fidelity, HIV testing seen as unnecessary. Where there is mistrust, testing is done to allay concerns of infidelity.	[33-35,44,45,50-52,54,65]
	Blame & partner reaction.	Fear of partner reaction, blame and straining relationships, which sometimes can lead to abandonment, divorce or even violence. Those who decide to test, especially without partner consent fear being held responsible for infidelity. Testing is therefore seen as a spoiler of harmony & trust in relationships.	[31,35,37,43,47,57,68]
	Fear of anticipated stigma & discrimination.	Fear of isolation, rejection & blame (for immoral behaviour) discouraging uptake of HIV testing.	[28-31,34,36,37,39,40,45,46],[48,49,51-56,60,61,63,65,66],[68,69]
		Being on ART also creates stigma-“responsible for spreading” HIV	[64]
Gender inequality & influence.	Gendered power relationships.	Female lack of negotiating power in marital relationship, including their lack of control over their health affects uptake of HIV testing. On the other hand, male domination of decision making, and their control over household resources enables them easily access testing.	[30-32,35,42,43,47,51,52,55],[57,59,68]
	Maintaining masculine identity.	Men also exhibit reluctance to test to preserve their masculine identity as strong and resilient. Others test in order to start treatment in order to maintain their status as breadwinners	[30,36,37,67,68]
Reproductive health aspirations.	Procreation & marital aspirations.	Larger social & reproductive health aspirations motivation for HIV testing. For women, testing invokes maternal duty the unborn child.	[29,34,40-44,46,47,52,54,58],[59]
		Desire to marry also prioritized; testing and being found HIV positive perceived as reducing chances of finding marital partner unless potential partner is also infected; others fear that sexual partners will shun then if they are HIV positive.	[34,36,61,67,69]
	Testing as marital requirement.	In some churches, it is a requirement for Christians to test before marriage could be sanctioned by the church. Individuals also wait till it is time to get married before seeking HIV testing.	[31,39,40,42,46,50,58,61]
Organisation/delivery of HIV services.	Opt-out HIV testing.	Routine offer of HIV testing to pregnant women & TB patients has shifted provider-service user power relationships in the testing process. Clients may fear being denied access to health care if they refused to test; sometimes women at antenatal care directed to bring their spouses for testing.	[29,30,35,41,53,54,57,58],[62]
	Location of HIV testing facilities.	Isolated testing centers within health facilities creates a barrier as people fear being seen seeking HIV testing as this may imply being sexually activity and/or already being infected.	[34,51,52,60,61,69]
	Feminisation of health care settings.	Health care facilities, particularly antenatal clinics perceived by men as female domains, out of bounds for men.	[35,52,57,67]
	HIV testing as package of health care.	Providing HIV testing with non-HIV related interventions provides an incentive to test as this helps mask HIV testing as primary objective in settings characterized by stigma. Provision of material benefits (i.e. food aid) to those found HIV positive also encourage uptake of HIV testing.	[43,63]
	Availability & efficacy of ART.	The availability of antiretroviral therapy & transformation of HIV into a manageable chronic condition has served as an incentive to test.	[30,55,59,65,66,69]
		However, the absence of/limited access to ART and the continued absence of a cure still inhibits uptake of testing.	[32,40,48,54,55,65,66,68]
	Burden of treatment for HIV-TB co-morbidity.	Patients with TB are subjected to (opt-out) HIV testing as part of care. However, dealing with the treatment burden of dual infections & double stigma forces them to deal with TB & HIV in succession rather than in concurrence, thus delaying uptake of testing.	[30,45,68]
Trust in the health system.	Quality of health care.	Perceived lack of confidentiality & privacy; perceived poor attitude of health staff affect health seeking behaviour. The use of non-familiar health personnel allays fears of breach of confidentiality, thus improving uptake of testing.	[28,29,33,34,40,43,46,49],[52,53,55,56,60,68]
	Distrust of testing process & technology.	Perceived unreliability of test results, notions of testing instruments as sources of infection & association of drawing of blood with rituals inhibit uptake of HIV testing. Confidence in test technology, including encouraging clients read test results creates credibility of test results.	[33,36,42,61]
	Conspiratorial beliefs.	HIV seen as a ‘plot’ by western countries to control/dominate SSA population & promote western interests.	[28,61]
Financial costs of HIV testing.	Indirect financial costs of HIV testing.	Long distance to testing sites & associated transport costs & travelling time discourage uptake of testing. Opportunity costs of travelling time, suspending livelihood activities & time of work inhibits HIV testing.	[33,36,42,50,52,56,57,63],[67]
	Direct financial costs.	Convenience of testing i.e. home/workplace-based testing at attenuates associated costs and travel time. Where user charges are non-existent, this encourages testing; paying for services competes with other human needs.	[33-35,39,42,47,49,50,56,57],[64]

Using the process of translation – transfer of ideas, concepts and metaphors across different studies [14], we compared the concepts of the papers i.e. paper 1 with paper 2 and the synthesised concepts of the two papers with paper 3 and so on, until all studies had been translated into each other [16,23]. The translation process was iterative to ensure that third-order constructs reflected concepts of the individual papers. New concepts were also identified through the process. Thus, we were able to re-interpret and re-conceptualise the findings to develop deeper meaning across the individual papers. The third-order constructs were transposed into a conceptual model showing the relationships between the different factors influencing uptake of HIV testing in SSA.

## Results

Forty-two (42) peer-reviewed qualitative and mixed-method papers published between 2001 and 2012 from thirteen (13) SSA countries were included in the synthesis (see Table 1). Thirty (30) were exclusively qualitative studies and twelve (12) were mixed-methods papers. While SSA is not a heterogeneous setting, some common characteristics could be deciphered: generalised HIV epidemic (HIV prevalence of more than 1% in the general population), and predominantly low-income countries with generally weak health systems and where HIV is mostly heterosexually transmitted. Twenty-three (23) second-order constructs were generated and summarised into eight (8) third-order constructs (Table 2). The findings are derived from second-order constructs and are categorised into enabling and deterring factors.

### Enabling factors for HIV testing

#### Poor health or death of sexual partner or child

Physical deterioration of health [30,32,37,39,40,43,46,48],[55,67,69] and poor health/death of sexual partner or child [31,43,48,59,68] elevated the perceived risk of infection and a decision to test:

*“My husband passed away two years ago, he had TB [tuberculosis]. I also looked after my daughter who was sick for a long time before she passed away and so my mind was not settled as I was suspecting that I could have acquired the deadly virus. When I heard that VCT was taking place today, I decided to put my mind at rest by being tested.”* (Female Tester, Zimbabwe) [43].

Experience of a sexually transmitted infection sometimes heightened risk perception [42,59] as did personal contact with or knowing someone who had died of AIDS [28,40,49], thus increasing the willingness to test. Experiences of multiple sexual partners and perceived partner unfaithfulness also created as sense of vulnerability and therefore encouraged uptake of HIV testing [32,48,59,60].

While heightened risk of HIV infection triggered uptake of HIV testing, sometimes it dissuaded people from testing as some assumed that they were already infected [36,40,55,62].

#### Availability of life-prolonging antiretroviral therapy

While HIV was previously perceived as a ‘death sentence’, the increasingly wider availability of life-saving medication in many countries of SSA has shifted this notion. In some countries of SSA, this was found to be an incentive to test [30,55,59,64-66,69]. Therefore, testing and knowing one’s HIV status was no longer associated with death, but as a path to start treatment and prolong one’s life. For pregnant women, and despite the reported existence of gender inequality in health seeking decision making, testing was often undertaken as maternal obligation to protect the unborn child from HIV infection [29,34,40-42,44,47,52,54,58].

*“I tested the time I was pregnant. I wanted to know if I was negative or positive, and I didn’t want to put my baby at risk…” (Woman respondent,* South Africa*)*[34].

#### HIV testing as preparation for marriage

In other instances, HIV testing was undertaken as an essential part of preparation for marriage [31,39,40,42,46,50,58,61]. For instance, a study in Ghana [50] encapsulates the role that the church was playing in increasing the uptake of HIV testing as an integral part of marriage preparation and counselling. As part of marital rituals, the church required prospective couples to seek HIV testing before the church could sanction such marriages. Explaining how this ‘mandatory’ strategy came about, one study participant noted that:

*“We had an HIV/AIDS awareness programme in our church…After an expert delivered a lecture, we were shocked at the numbers… we decided all those who are planning to marry must be tested for HIV/AIDS… We wanted to protect our future generations…”* (Respondent, Ghana) [50].

#### Organisation and delivery of HIV testing

The roll-out of diverse HIV testing initiatives has contributed towards uptake of HIV testing. The implementation of ‘opt-out’ provider-initiated HIV testing - HIV testing and counselling which is recommended by health care providers to persons seeking health care services as a standard component of medical care - has contributed to increased uptake of HIV testing. For instance, service users reported being tested at antenatal care [41,54,57,58,62], or as TB patients [30,45,68] as an integral part of health care. This strategy was sometimes regarded as non-voluntary. Individuals sometimes acquiesced to the pressure by service providers to test:

*“Although they say its voluntary, but they put pressure on you to test for it….If you don’t want to do it then, they must say ‘Okay, tell us when you feel comfortable for an HIV test.”’* (23-year old female tester, South Africa*)*[30].

Similarly, bringing testing services closer to the people through outreach mobile HIV testing attenuated (opportunity) costs of travelling and waiting times [33-35,49], and provision of free testing services in Kenya [63], and expectations/provision of material benefits (i.e. food relief) encouraged uptake of HIV testing [43,63,67]. The use of non-familiar counsellors through mobile VCT was viewed as enhancing confidentiality, thus improving uptake of HIV testing [33,43,60].

#### Social network influence and support

Decision making about HIV testing were inextricably linked to social network influence [36-38,40,42,49,56-58,67]. A study in Zambia [38] poignantly describes how individuals sought the views and support of their peers and family members during the decision making process about seeking HIV testing. In part, this was because friends and family members were crucial sources of psychosocial support and for family members a critical source of economic support:

*“In the first place I never wanted to go there [for an HIV test], but I consulted my sister. She said no and I also said no. But afterwards I asked my brother who said . . . you should go for VCT, so that is when I went.”* (Male tester, Zambia) [38].

### Deterrents to HIV testing

#### Perceived low risk of infection

One recurring theme was that across SSA, individuals self-assessed their risk of infection. Lay interpretations of being at low risk of infection [43-45,49], sometimes because of abstaining from sex or lacking a sexual partner [32,33,42,51] negatively affected uptake of HIV testing. Proxy testing - adopting the status of sexual partner - was sometimes used as a risk estimate and a representation of one’s own HIV status [38,60]. Some people felt they were at low risk of infection because they trusted their partner [44,45,50-52,65] or because HIV was mainly perceived as a problem for sex workers [39-41]. A lack of physical symptoms or deterioration of health was also perceived as a sign of not being infected [33,34,65,69]. In low prevalence settings like Mali [28], not knowing someone with HIV or who had died of AIDS created a perception of being at low risk of infection, thus undermining uptake of HIV testing.

#### Stigma, social exclusion and gendered relationships

Fear of stigma was another dominant theme for the low uptake of HIV testing in many settings of SSA [28-32,34,36-40,45,46,49,51-55,60],[61,63,67-69]. Particularly because HIV transmission is predominantly heterosexual across SSA, being seen at a testing centre was synonymous with sexual promiscuity and assumed HIV-positive status [34,51,52,60,61]. For TB patients in South Africa, Ethiopia and Cameroon [30,45,68], the prospects of dealing with potential ‘double’ TB/HIV stigma acted as disincentive to test. The fear of HIV-related stigma is reflected in the following:

*‘Even if I am already infected, nobody knows and it causes me no problems, at least for now. Imagine I go and do the testing and I find out I am positive, for how long will I hide it? Once people get to know I will be finished. My family will shun me. My friends will desert me. I will not be able to get a decent job. That is dying even before the infection kills me.’* (25-year old female non-tester, Nigeria) [39].

A study in Tanzania [64] found that while the effect of ART on improving corporeal health had motivated uptake of HIV testing, a new form of stigma had emerged in which individuals on ART were stigmatised as they were viewed as being responsible for the continued spread of HIV on account of them living longer with HIV. This in turn undermined uptake of HIV testing.

Across the sub-regions of SSA, fear of social exclusion also negatively influenced uptake of HIV testing [37,38,42,49,56,58,64,67]. The fear of losing social support [38-40,42,64] and sexual partners [36,37,64,67], and the fear of straining marital relationships, including possibilities of abandonment, divorce, or even violence [35,43,68,69] inhibited uptake of HIV testing. The desire to marry also weighed heavily on people’s mind and a positive sero-status was viewed as threatening the chances of finding a marriage partner [34,61,67,69]. In Uganda, there was fear that where discordance arose, test results could be used as confirmation of infidelity which could strain marital relationships [52,62].

Within marital relationships, gender inequality affected women’s uptake of HIV testing. Studies in South Africa, Uganda and Tanzania found that men enjoyed decision-making autonomy on HIV testing [30,42,57]. Most studies reported women lacking control over their health; decisions about seeking HIV testing had to be discussed with, and permission obtained from, spouses [30-32,35,43,44,47,51,52,55],[57,62,68]. In Tanzania and Zimbabwe, obtaining consent still raised suspicions of possible infidelity [31,43] and those found HIV positive risked being blamed for contracting HIV [37,47,57,68]. Thus HIV testing was shunned to avoid straining marital relationships. In Malawi, Uganda and Zimbabwe, men refused to test since this was at odds with masculine identity of self-confidence, resilience and stoicism [36,67]. In Zambia, one study found that if the wife suggested testing, this was viewed by men as undermining their role as decision makers [37].

#### Quality of HIV testing services

Across sub-regional settings of SSA, individual perceptions of and experiences with the health care system undermined uptake of HIV testing. Perceived lack of confidentiality by health staff [29,32,40,43,46,49,55,60],[68]; perceived lack of confidence in the competence of health personnel [28,29,40]; and perceived poor attitude of health staff [52,53] dissuaded people from testing. Perceived unreliability of test results in Malawi and Uganda [33,36,42] and distrust of HIV testing technologies in Uganda and Zambia [42,61] discouraged uptake of HIV testing. Describing his concerns about confidentiality, one respondent in Malawi said:

*“It’s because if I can be tested at Mhojo Health Centre, VCT counsellors there know me and if that counselor at the VCT [centre] finds me with the virus then he can start spreading the messages to friends of mine, and if I know about that then it becomes very bad to my life, that’s why to be tested with someone else whom you never know it’s good”* (Male, 48 years old, Malawi) [33].

Similarly, at health facilities, perceived poor location of testing facilities undermined uptake of HIV testing [34,51,52,60,61,69]. Secluded testing facilities or use of VCT-specific clinic cards created social visibility of seeking HIV testing and assumption of being sexually active and/or already being infected. Where couple counselling was conducted at antenatal clinics, men perceived the testing sites as feminised settings, and therefore out of bounds [30,35,52,57,67].

#### Trust in the health care system and conspiratorial beliefs

Although not universally held across SSA, conspiratorial beliefs about HIV being a ‘western plot’ to dominate SSA were reported in Mali and Zambia [28,61]. This inhibited uptake of HIV testing. These conspiracy views were nested within historical discourse about “colonial projects [which] turned African patients into objects to be studied and scrutinised, categorised and measured” [61]. In Mali [28], HIV was viewed as an invention to halt the growth of the African population or to sell western bio-medical products. Promotion of HIV testing by western countries as a gateway to accessing treatment and living a longer, healthy life was therefore viewed as a ploy to expand western countries’ interests:

“*In reality, AIDS is an invention to sell condoms. The West created the idea of AIDS to put a stop to sexual relations or even better it’s a policy to put a brake on the growth of the African population*.”(23-year old male non-tester*,* Mali*)*[28].

While HIV testing services were increasingly provided free of charge in many settings of SSA, this ‘gift’ by western countries and agencies or their local affiliates was viewed as attempts to further subjugate the weak African populations and to benefit western countries. As one old man in Zambia put it:

*“Look around you, who is making money off of this disease? It is not Zambians. It is you [white Westerners]. This is why people are suspicious of this disease. This is why they think it [AIDS] was brought in from the outside.”* (Male respondent, Zambia) [61].

Where religious discourse was dominant, a study in a rural setting of Zambia found that distrust of drawing blood for Satanic motives (synonymous with vampires), and perception of western medical technologies as instruments of the ‘devil’ created apprehension about HIV testing [61]. HIV testing was viewed as plunging an individual into ‘spiritual darkness, pain, loneliness and death’ [61].

#### Financial costs of accessing HIV testing

In the context of fragile livelihoods, the direct and indirect financial costs of accessing testing services inhibited uptake of testing [33,36,42,50,52,56,63]. Although HIV testing had become increasingly free in many settings of SSA in order to improve access levels, where user fees were charged, individuals weighed the benefits of testing against other competing human needs [33,35,39,42,47,50]. More so, the indirect opportunity costs of suspending income generating activities and time-off work discouraged uptake of HIV testing [42,50,57,67]. One respondent in Nigeria said:

*“It costs 1000 Naira (approximately 8 US Dollars) to do blood test for HIV in the laboratories in Enugu. That is the minimum you can get it. I even heard that it costs more than that in some laboratories. So, why would I spend that amount of money to find out if I am HIV positive or not?”* (24-year old non-tester, Nigeria) [39].

#### Perceived psychological burden of living with HIV

The motivation to test also depended on a person’s perceived ability to manage HIV. Even with availability of life-prolonging treatment, in many settings of SSA, a positive-HIV test result was still associated with death [29,33,34,38,40,42,43,47],[48,54,55,60,65,69] and mental distress was anticipated [32,34,36,61,69] with an HIV positive test causing an individual to ‘begin to think too much.’ This was perceived as hastening physical deterioration of health:

*“Why look for troubles; I will never do a test. I cannot look for my death. I am afraid of dying. Haven’t you seen those who go for counselling? They are the ones who die very soon*.”(Male non-tester, Tanzania) [47]

The reported absence of, or limited access to, treatment in some settings was a disincentive to test [32,40,48,54,55,65,66,68]. Similarly, given the prevalence of HIV-TB co-infection, TB patients in Cameroon and South Africa avoided HIV testing to avoid the burden of being on dual strong treatment regimens for two diseases [30,68].

#### Discussion: synthesis and line-of-argument

**Figure 2:**
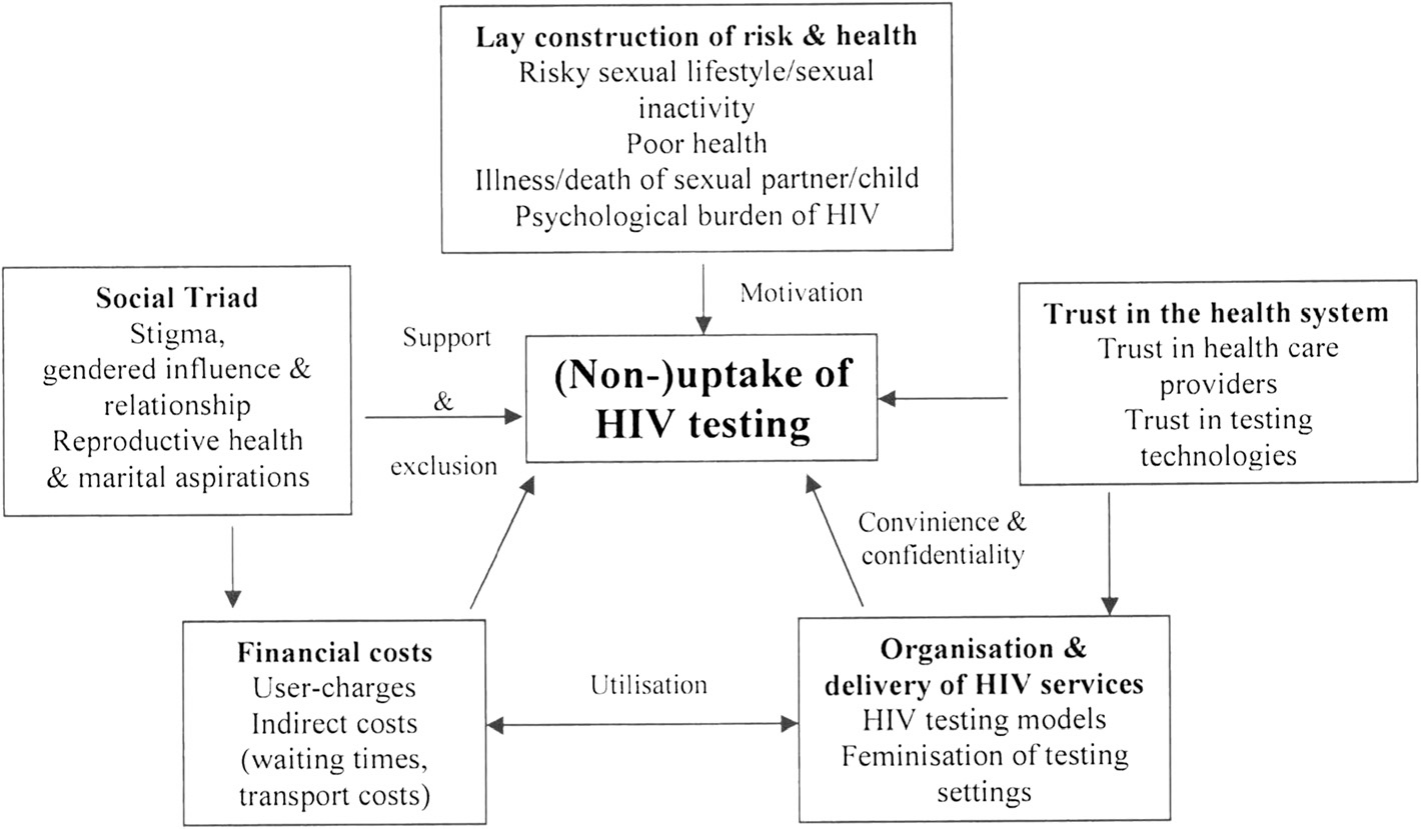
Conceptual model of third-order constructs of nested relationships of factors influencing uptake of HIV testing.

This synthesis shows that uptake of HIV testing in SSA is influenced by an array of individual, relational and contextual-based factors. While SSA is not a homogeneous setting, our synthesis suggests that the barriers and facilitators are comparable across SSA. It is worth pointing out that the factors influencing uptake of HIV testing are not mutually exclusive. As depicted by our conceptual model (Figure 2), they are inextricably linked and may coalesce or reinforce one another to influence uptake of HIV testing. Based on the analysis and interpretation of second-order constructs, high-level third-order constructs were developed (Table 2) to form a ‘line of argument’ about factors influencing uptake of HIV testing in SSA. These are condensed into four themes (Figure 2) and discussed as below:

#### Lay construction of risk of infection and health

First, one dominant factor that influences uptake of HIV testing is lay construction of risk and health. Individuals engage in intense activity of experience-sorting and interpretation as they situate themselves in terms of danger [70]. This lay assessment is informed by individuals’ knowledge of own and partner’s sexual behaviour and observations and experiences of their health [71]. This lay analysis influences behaviour in two different ways. While heightened risk of infection provides impetus to test, there is also a disjunction between perceived risk of HIV infection and uptake of testing, as those who perceive themselves as being infected already do not see the value of knowing their HIV status. Thus, HIV testing is more often undertaken when there is clear decline in health status which necessitates access to health care.

Related to physical health is the psychological burden of living with HIV. Despite the increasingly wider availability of antiretroviral therapy in most parts of SSA, its impact on uptake of HIV testing still remains mixed. This is because while treatment is saving lives and has ‘normalised’ HIV from a fatal to a chronic condition, its incurable nature reduces the motivation to test (Figure 2). Knowing one’s HIV status is viewed as imposing an inordinate psychological burden and is associated with imminent death. HIV testing is therefore undertaken when it is an absolute necessity – when diagnosis is needed to access health care [69].

#### Trust in the health system and conspiratorial beliefs

Second, even when individuals view themselves at risk of HIV infection and/or are willing to seek HIV testing, uptake of testing is influenced by people’s trust in the health care system and providers (Figure 2). The lack of trust manifests itself in lack of confidence in individual health workers and trust in the health institution as a whole [72]. Perceived poor quality of health services as characterised by inability by health workers to maintain confidentiality, perceived poor calibre of health workers and lack of trust in testing technologies inhibit uptake of testing. As Gilson has noted, health systems are social institutions and therefore people’s perceptions of and experiences with the health care system is crucial in influencing service utilisation which even good technical care may not remedy [73]. Studies on trust conducted in the United States of America have reported how distrust of health providers and health system as a whole affect utilisation of HIV services [74-76]. For instance, one study reported that trust in physicians was associated with acceptance of ART and a minority of individuals that felt mistreated by health care providers were resistant to accepting ART [74]. Narratives from the synthesis indicate that lack of trust in health care providers was attenuated by the provision of HIV testing through non-facility based HIV testing by non-familiar providers, thus improving uptake of HIV testing (Figure 2).

Another dimension of lack of trust in the health system relates to conspiracy narratives (Figure 2). In a few settings of SSA, HIV and HIV testing were viewed as western countries’ insidious ways of dominating SSA. HIV testing was viewed as being used to benefit western countries through creation of market for bio-medical products [28] and job opportunities for its citizens [61]. These geo-political conspiratorial beliefs sometimes coalesce with religious discourse. In Zambia for instance, blood drawn for HIV testing was viewed as being used for satanic rituals [61,77]. These findings corroborate previous medical research conducted in Gambia and Zambia where local people were highly suspicious of and shunned medical tests which involved the drawing of blood [78,79].

#### Social triad: Stigma, gendered influence and reproductive health aspirations

Third, HIV testing behaviour is strongly socially delineated. Social relationships play a significant role in influencing HIV testing behaviour through social influence and perceived (lack of) social support (Figure 2). For instance, in the absence of strong formal safety nets, social capital is central to survival. Therefore, the desire to preserve social relationships and identity inhibit uptake of HIV testing. This is because while individuals may acknowledge the importance of knowing their HIV status and even show willingness to seek testing in response to (perceived) decline in health or because of previous sexual risk behaviour, ultimate decision making and attitude towards testing is influenced by concerns about anticipated stigma, which is sometimes inextricably linked to possible loss of economic support. Thus, being found HIV positive represents an undesirable characteristic, a ‘spoiled identity’ [80] from which individuals try to distance themselves.

Paradoxically, while the availability of treatment has become an incentive to test, a new form of stigma has emerged. A study in Tanzania [64] found that while ART roll-out had led to the ‘normalisation’ of HIV and thus stimulated uptake of testing, the stigmatisation of people on treatment that they ‘spread the disease’ also undermined uptake of HIV testing.

Gendered power relationships also undermine uptake of HIV testing (Figure 2). In most parts of SSA, ultimate authority on health care seeking lies with men [81,82] and communication with, and support from, partners improves uptake of HIV testing by women [83,84]. This was a common narrative amongst women in some of the synthesised papers. Their lack of access to and control over financial resources affected their access to and utilisation of HIV testing services. Conversely as primary caregivers, their subordinate role in decision making about HIV testing was mitigated by their regular contact with reproductive and child health services, thus being able to utilise HIV testing services. The onset of provider-initiated HIV testing also absolved women from blame for testing without their partners’ consent by shifting attention to testing as part of routine health care.

Similarly, the uptake of HIV testing is inextricably linked to individuals’ marital and reproductive health aspirations. Thus, marriage and parenthood represent social duties, expectations and individual aspirations [85], and a connection with one’s community [86,87]. This affects uptake of HIV testing behaviour in two opposing ways. As an enabler, both men and women sought HIV testing as preparation for marriage or achieving reproductive health aspirations. Those that had never tested claimed willingness to seek HIV testing when it was time to get married. For women, as Fortes has put it, “the achievement of parenthood is regarded as a *sine qua non* for the attainment of the full development as a complete person to which all aspire....and a woman becomes a woman when she becomes able to bear children and continued child bearing is irrefutable evidence of continued femininity” [86].

Narratives from the synthesised papers suggest that HIV testing was accepted during antenatal care primarily because it was essential for achieving reproductive health aspirations and as a moral and social obligation to give birth to a healthy child. On the other hand, both men and women declined HIV testing for fear of straining marital relationship or undermining chances of finding a marriage partner.

#### Organisation and delivery of HIV testing: mitigating the financial and social costs

Lastly, the synthesis shows that uptake of HIV testing is influenced by the way HIV services are delivered. Where user-fees are charged or services are far away, investment in health (HIV testing) competes with, and is ranked low in relation to, other immediate human needs. This is because, for people in precarious living conditions, access to, and utilisation of, health care imposes inordinate opportunity costs [81]. However, the roll-out of different HIV testing initiatives such as mobile HIV testing services, provider-initiated HIV testing, home-based HIV testing [88] has mitigated these barriers such as distance, financial costs, long waiting times, inconvenient testing hours, and allayed fears of perceived lack of confidentiality [10,89,90]. When individuals are in contact with the health system for other health conditions, provider-initiated HIV testing ensures uptake of testing not only because it is necessary and is conveniently available at the time of seeking medical attention, but also because it helps preserve service users’ sense of moral worth by not making assumptions about their behaviour which could lead to stigmatisation [91]. The drawback is that men shunned testing services even if they were readily available if they viewed them as being provided in settings perceived as ‘female spaces’ such as antenatal clinics [92].

#### Strengths and limitations

The strength of this synthesis lies in the extensive search of literature. The inclusion of papers utilising different methodological approaches, including mixed-methods papers provided in-depth insight into factors that influence uptake of HIV testing. Also, like previous studies that have used the meta-ethnography approach [17,20,22,25,26], not using pre-determined quality assessment criteria or excluding papers on the basis of pre-specified quality assessment criteria enabled us to draw on the ‘richness’ of these papers. Using a multi-disciplinary team also enriched the synthesis by enabling us to draw and collate team members’ interpretations of the findings. However, an inherent weakness of this synthesis is the possibility of having missed some publications. We tried to mitigate this by scouring references of selected papers and manually searching the data bases. Another limitation of this synthesis is that due to language constraints, we only included papers published in English. Similarly, due to publication restrictions, the context of the synthesised studies were not extensively described thereby limiting detailed contextualisation of the synthesised findings and comparing the findings across different settings.

## Conclusions

Uptake of HIV testing in SSA is influenced by an array of often inter-linked factors. Despite the heterogeneity of SSA, our findings suggest that there is a strong similarity in the barriers to and facilitators of HIV testing across SSA. Lay interpretation of risk of infection either encourages or discourages uptake of HIV testing. Depending on past sexual lifestyles and the state of individual, marital partner and child’s corporeal health, individuals construct own probabilities of being infected. While direct and indirect financial costs inhibit uptake of HIV testing, access to HIV testing is also deeply engendered, and individuals also have to balance the social benefits and costs of seeking HIV testing. Although the wider availability of HIV testing and treatment services and roll-out of various HIV testing strategies has contributed towards increased uptake of HIV testing, lack of confidence in the health system and conspiratorial beliefs undermine testing uptake. Even though the enablers and barriers to uptake of HIV testing cut across many settings of SSA, interventions aimed at increasing uptake still need to be context specific, sustaining the enabling factors and concurrently addressing the barriers.

### Policy and practical implications

The synthesis suggests that the policy of provider-initiated HIV testing coupled with increased wider availability of life-saving HIV medication is crucial in scaling up uptake of HIV testing in SSA. Due to fear associated with seeking HIV testing, availability and convenience of provider-initiated HIV testing provides that extra ‘push’ that enables individuals to overcome barriers and effect their intentions to test and at the same time assuage fear of stigma and attenuate costs. This, therefore, calls for stepping up provider-initiated HIV testing when individuals come into contact with the health system.

At practical level, our synthesis suggests the need for scaling up and sustaining the roll-out of different and locality-specific HIV testing models i.e. mobile HIV testing to respond to the peculiarities of each setting, even within the same country. Improving quality of HIV service delivery, particularly ensuring confidentiality - which many studies identified as a barrier - is also vital. Interventions such as home-based HIV testing that focus on social network relationships (i.e. couples and households) rather than individual-focused interventions are also critical given inequitable power dynamics and the significance of social networks in decision-making processes about HIV testing. Such strategies could also help assuage fears of confidentiality as reported in many studies as well as attenuate direct and indirect financial costs of seeking HIV testing. Given the reported persistence of stigma, continued sensitization campaigns are need. Also, provision of HIV testing interventions particularly in non-clinical settings need to be combined with screening for other less stigmatizing health conditions to avoid stigma associated with being seen accessing HIV testing. Most crucially too, through sensitization campaigns, there is need to focus on addressing socially constructed individual risk assessments, especially in low HIV prevalence settings where HIV may be viewed as unreal and a far-off threat. In settings where mistrust and conspiratorial beliefs about HIV and HIV testing exist, these need to be addressed through sensitization campaigns.

## Competing interests

The authors declare that they have no competing interests.

## Authors’ contributions

MM, VB and SM conceptualized the study. MM, SG and OM did the literature search. MM and HN did the title and abstract review and all authors were involved in full-text review. MM drafted the first manuscript into which co-authors contributed input. All authors have given final approval of the version to be published.

## Pre-publication history

The pre-publication history for this paper can be accessed here:

http://www.biomedcentral.com/1471-2458/13/220/prepub
